# The Dual Roles of Human γδ T Cells: Anti-Tumor or Tumor-Promoting

**DOI:** 10.3389/fimmu.2020.619954

**Published:** 2021-02-16

**Authors:** Yang Li, Gen Li, Jian Zhang, Xiaoli Wu, Xi Chen

**Affiliations:** ^1^ School of Chinese Materia Medica, Tianjin University of Traditional Chinese Medicine, Tianjin, China; ^2^ School of Life Sciences, Tian Jin University, Tian Jin, China; ^3^ College of Pharmaceutical Engineering of Traditional Chinese Medicine, Tianjin University of Traditional Chinese Medicine, Tianjin, China

**Keywords:** γδ T cells, tumor, human, immunity, immunotherapy

## Abstract

γδ T cells are the unique T cell subgroup with their T cell receptors composed of γ chain and δ chain. Unlike αβ T cells, γδ T cells are non-MHC-restricted in recognizing tumor antigens, and therefore defined as innate immune cells. Activated γδ T cells can promote the anti-tumor function of adaptive immune cells. They are considered as a bridge between adaptive immunity and innate immunity. However, several other studies have shown that γδ T cells can also promote tumor progression by inhibiting anti-tumor response. Therefore, γδ T cells may have both anti-tumor and tumor-promoting effects. In order to clarify this contradiction, in this review, we summarized the functions of the main subsets of human γδ T cells in how they exhibit their respective anti-tumor or pro-tumor effects in cancer. Then, we reviewed recent γδ T cell-based anti-tumor immunotherapy. Finally, we summarized the existing problems and prospect of this immunotherapy.

## Introduction

γδ T cells are the non-classical cell subgroup characterized by expression of γδ heterodimeric T cell receptor (TCRγδ) on cell surface. They only account for 1% to 5% of T lymphocytes in peripheral blood circulation and lymphatic circulation, and predominantly reside in the mucosal tissues such as skin, intestine, lung, and uterus (1–3). γδ T cells are the intermediate group of cells between innate and adaptive immune cells, serving as a bridge between innate immunity and adaptive immune response (4, 5). They play important roles in tumor immunity. Depending on the microenvironment, different γδ T cell subgroups can have anti-tumor or pro-tumor activities.

Compare with αβ T cells, γδ T cells have different antigen recognition mechanisms and capabilities without the histocompatibility complex (MHC) and the second signal (CD28 and CD80/86) (6). They can use TCRγδ and natural killer cell receptors (NKR) to recognize a variety of tumor-associated antigens (TAA), including non-peptidic prenyl-pyrophosphate antigens (PAg) and stress proteins (7). The PAg are products of isoprenoid biosynthesis pathways, such as isoprene pyrophosphate (IPP) from mammalian cells and (E)-4-Hydroxy-3-Methylbut-2-Enyl Diphosphate (HMBPP, the strongest stimulant of γδ T cells) from pathogenic microorganisms (8–12). Besides, the stress proteins will up-regulate or ectopically express under the stress conditions, such as apolipoprotein A1-F1-ATPase complex (F1-ATPase Apo A1) (13), MHC-like molecules MICA/B, UL16 Binding protein (ULBP) (14–18), endothelial cell protein C receptor (EPCR) (19, 20), heat shock protein (21–23) and human MutS homolog 2 (24–26). These antigens can activate γδ T cells to secrete interferon γ (IFN-γ) and tumor necrosis factor α (TNF-α) (6), or kill tumor cells through Fas/FasL and antibody-dependent cell-mediated cytotoxicity (ADCC) (27–30). Moreover, γδ T cells can also enhance the anti-tumor ability of other immune cells by secreting cytokines or expressing costimulatory molecules. For example, human γδ T cells can stimulate the cytotoxicity of NK cells through expressed the costimulatory molecule CD137L (31). γδ T cells have been used in clinic for the treatment of non-small cell lung cancer and breast cancer. Such γδ T-based immunotherapy appeared to be safe and well-tolerated in patients (32–35).

However, it was reported that γδ T cells could also promote cancer development (36). For example, as one of the main sources of interleukin-17 (IL-17), tumor-infiltrating γδ T cells were shown to promote tumor development and metastasis by enhancing angiogenesis and recruiting inhibitory cells (37–40). Tumor-infiltrating γδ T cells could also directly induce the apoptosis of anti-tumor immune cells (41).

In this review, we introduced the classification of human γδ T cells and summarized how γδ T cell subsets play different roles in tumorigenesis. We further discussed the γδ T cell-based anti-tumor immunotherapy which has been widely used in clinic. Finally, we briefly summarized the current limitation and caveats associated with such therapy, and proposed new approach for optimization. We believe that the summary of biological functions of different γδ T cells can help us improve our understanding of tumor microenvironment, and provide novel insights for anti-tumor immunity.

## Classification of γδ T Cells

Human γδ T cells can be classified into different groups based on the expression of TCRγ chains or TCRδ chains, and they can be further classified by the expression of different CD molecules (42, 43).

### Classification Based on the Expression of TCRγ Chain or TCRδ Chain

Different TCRγ chains (Vγ2/3/4/5/8/9) and TCRδ chains (Vδ1/2/3/5) can be combined to form different types of γδ T cells. Interestingly, each TCRδ chain usually forms with one or several dominant TCRγ chains a fixed combination pattern, rather than with random combinations (44–47).

Different γδ T cells have diversified distribution and functions. Vδ1 chain can interact with different γ chains to form various γδ T cells. They are mainly distributed in the skin, intestine, liver, spleen and mucosal tissues. The role of Vδ1 T cells is controversial. In certain situations, they have been shown to have strong anti-tumor effects in colorectal cancer, multiple myeloma, chronic lymphocytic leukemia (48–50). On the other hand, tumor-infiltrating Vδ1 T cells often demonstrate strong immunosuppressive effects. They secreted IL-17 and transforming growth factor-β (TGF-β) (51), expressed programmed cell death 1 ligand 1 (PD-L1), and inhibited the activation of other immune cells (41).

The Vδ2 chain only combines with the Vγ9 chain to form the Vγ9Vδ2 T cells, which mainly exist in peripheral blood. Given that the Vγ9Vδ2 T cells have strong anti-tumor effects in various types of tumors, they were widely used in clinics (52–55). In addition, they have also been shown to kill the cancer stem cells (CSC) in various tumors including colon cancer, ovarian cancer, and neuroblastoma (56, 57).

The Vδ3 chain mainly interacts with the Vγ2 and Vγ3 chains. Vδ3 T cells mainly exist in the liver, and also in a small amount in the peripheral blood of patients with chronic lymphocytic leukemia. The functions of Vδ3 T cells in tumors have not been elucidated in depth (58–61).

The Vδ5 chain usually combines with the Vγ4 chain to form the Vγ4Vδ5 T cells. They mainly exist in peripheral blood. The TCR of Vγ4Vδ5 T cells could directly bind to the endothelial protein C receptor (EPCR) to recognize epithelial tumor cells. Like Vδ3 T cells, they were rarely studied for their tumor-related functions (19, 62) (**Table 1**).

**Table 1:** Subsets of human γδ T cells.

Subset	Paired TCRγ chains	Cellular localization
Vδ1	Vγ2, Vγ3, Vγ4, Vγ5, Vγ8 and Vγ9	Skin, intestine, liver, spleen and mucosal tissues
Vδ2	Vγ9	Peripheral blood
Vδ3	Vγ2, Vγ3	Liver and peripheral blood
Vδ5	Vγ4	Peripheral blood

### Classifications Based on the Phenotype of CD Molecules

Human γδ T cells can be classified based on the expression of CD27 and CD45RA. The naive type (T_naive_, CD27^+^CD45RA^+^) and the central-memory phenotype (T_CM_, CD27^+^CD45RA^-^), mainly exist in the secondary lymphoid organs. T_CM_ can maintain immune memory for a long time and quickly mediate immune response after receiving antigen stimulation. The effector-memory type (T_EM_, CD27^-^CD45RA^-^) and terminally-differentiated type (T_EMRA_, CD27^-^CD45RA^+^) mainly exist at the site of inflammation and exert instant effects, namely secreting cytokines and exerting cytotoxicity (63, 64).

### Classification of γδ T Cells According to Their Cellular Function

Based on their varied functions, γδ T cells can be divided into several subtypes. Similar to αβ T cells, effector γδ T cells can exert an anti-tumor effect through various pathways. Regulatory γδ T cells (CD4^+^CD25^+^Foxp3^+^) or inhibitory γδ T cells can regulate the immune balance and maintain immune tolerance (17, 51). In addition, γδ T17 cells can produce IL-17 to promote tumor development (6, 36, 41).

## γδ T Cells Play a Direct Anti-Tumor Role

### The Tumor-Associated Antigens Recognition by γδ T Cells

Vγ9Vδ2 T cells recognizes TAA through TCRγδ and NKR and Vγ9Vδ2TCR can recognize PAg. This type of antigen was a product of isoprenoid biosynthesis pathway in eukaryotic cells, such as IPP and the adenylated, thymidylated, and uridylated triphosphate derivatives. In tumor cells, the isoprenoid biosynthetic pathway is enhanced to ensure energy supply and PAg accumulation, prompting recognition by the Vγ9Vδ2 T cells (65–68). Vγ9Vδ2TCR requires the help of Butyrophilin (BTN) 3A1 to recognize tumor cells. BTN3A1 is an immunoglobulin-like molecule with immunomodulatory function, which could mediate the interaction between γδ T cells and PAg, and could also be directly recognized by Vγ9Vδ2TCR (69–71). There were two theories on how BTN3A1 helps Vγ9Vδ2TCR recognize PAg. The first proposed mechanism was that BTN3A1 is a sensor that senses the level of PAg inside the cell. The intracellular B30.2 domain of the BTN3A1 molecule is a positively charged pocket that could directly bind to PAg, lead to changes in the structure of the extracellular dimer of BTN3A1 that can be recognized by Vγ9Vδ2TCR, and then activate the γδ T cells (72–77). The second proposed mechanism was that BTN3A1 formed a BTN3A1-PAg complex with PAg, presented PAg to the outside of the cell, and directly bound to Vγ9Vδ2TCR to activate γδ T cells (78). The latest study found that BTN2A1, which was in the same family as BTN3A1, was also a ligand for Vγ9Vδ2TCR and necessary for Vγ9Vδ2 T cells to recognize PAg. BTN2A1 and BTN3A1 can be found on the surface of tumor cells and recognized by two sites of Vγ9Vδ2TCR. BTN2A1 is recognized by the Vγ9 area, and BTN3A1 is recognized by the Vδ2 area (79, 80). In addition, Vγ9Vδ2TCR could recognize the F1-ATPaseApoA1 complex. This complex are normally expressed in the inner membrane of mitochondria, but some tumor cells, such as human leukemia (K562) cells and lymphoma (Raji) cells, could ectopic express it on the cell membrane. ApoA1 in the complex could not directly activate Vγ9Vδ2 T cells, instead it plays a function in stabilizing the interaction between Vγ9Vδ2TCR and F1-ATPase (13, 81).

Vγ9Vδ2 T cells could also recognize TAA through NKR, such as the natural killer 2D receptor (NKG2D) and DNAX accessory molecule 1 (DNAM-1). NKG2D is a lectin-type activation receptor, expressed on most natural killer cells (NK) and natural killer T (NKT) cells and partly expressed on γδ T cells and antigen-activated CD8^+^ T cells (82). When γδ T cells contacted by the tumor cells, Vγ9^+^ subpopulations rapidly proliferated, and γδ T cells up-regulated their NKG2D expression (83). NKG2D ligands on tumor cells include MICA, MICB and ULBP1~4 (84, 85). They could be recognized by NKG2D and enable γδ T cells to exert anti-tumor function (82). DNAM-1 is expressed on the γδ T cells and believed to promote the secretion of cytokines and enhance the cytotoxicity of immune cells. Vγ9Vδ2 T cells used DNAM-1 to recognize Nectin-2 and PVR, which were widely expressed on the tumor cells (86–88). Shielding DNAM-1 from the surface of γδ T cells could significantly inhibit its ability to kill tumor cells (89). It was shown that DNAM-1 is one of the important factors mediating γδ T cells to recognize tumor cells.

Vδ1 T cells also recognize tumor cells through TCRγδ and NKR. Vδ1TCR could recognize MHC-like molecule CD1d and the lipid antigen presented by it (90, 91). CD1d is expressed on a variety of cancers, such as myeloma, breast cancer and prostate cancer (92–94). The decrease of CD1d molecules on the primitive neuroectodermal tumor cells would cause these cells to evade immune recognition (95). In addition, Vδ1TCR could recognize tumor cells through MICA, but the MICA bindings by Vδ1TCR and NKG2D were mutually exclusive (96). Vδ1 T cells also express NKR. These cells recognize ULBP3 which is expressed on chronic lymphocytic leukemia of B-cell type (B-CLL) through NKR (97). They recognize human breast cancer cells through NKG2D, significantly preventing the disease progression (35). In addition to NKG2D and DNAM-1, Vδ1 T cells stimulated by IL-2 or IL-15 also express NKp30, NKp44 and NKp46 (48, 98), and have strong IFN-γ secretion ability (99, 100). Moreover, it has been confirmed that in acute myeloid leukemia, the ligand of NKp30 is B7-H6, a member of the B7 family (101).

**Figure 1:**
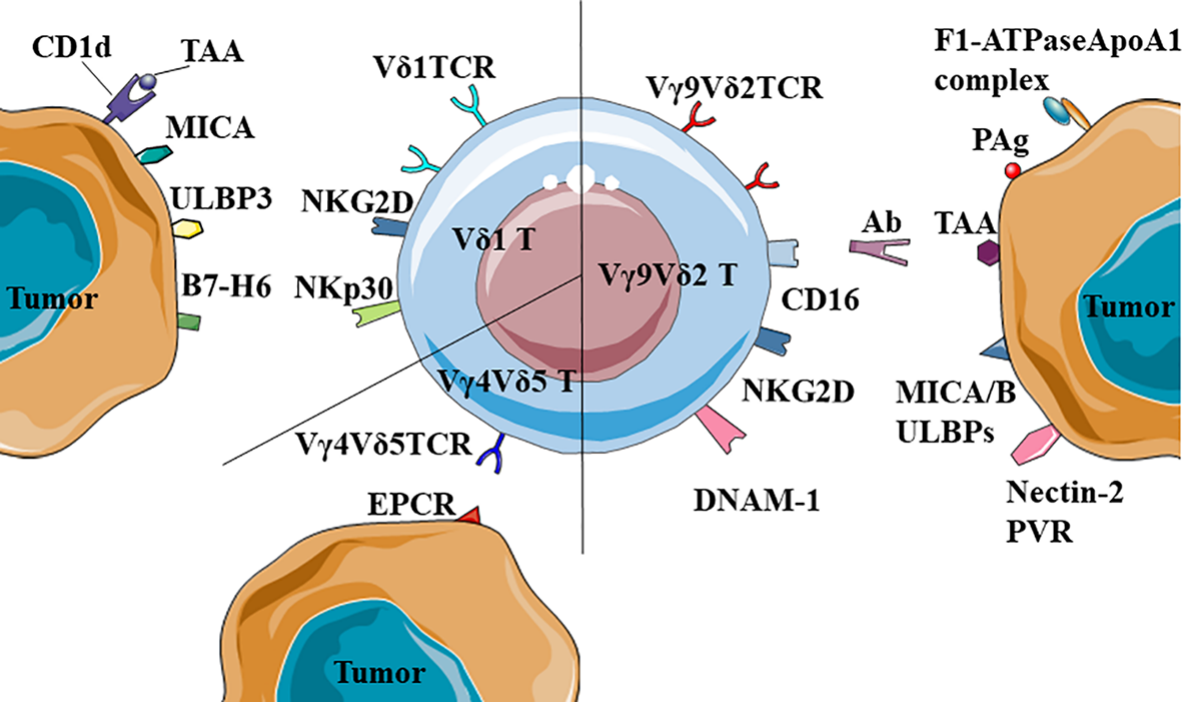
Recognition of tumor-associated antigens (TAA) by different γδ T cells. Vδ1TCR could recognize MICA or the complexes of CD1d and TAA; NKR+ (NKG2D and NKp30) Vδ1 T cells could recognize ULBP3 and B7-H6. Besides, Vγ9Vδ2 T cells could also recognize and kill tumors by CD16-mediated antibody-dependent cell-mediated cytotoxicity (ADCC). Notably, Vγ4Vδ5TCR could recognize the antigen of epithelial tumor cells, EPCR.

Other studies have also confirmed that Vγ4Vδ5TCR can recognize EPCR, which is expressed on the epithelial tumor cells (19, 20) (**Figure 1**).

### Anti-Tumor Mechanism of γδ T Cells

First, γδ T cells could kill tumor cells directly through secreting perforin and granzyme B (82). γδ T cells recognize tumor cells and release perforin and granzyme B into the synaptic space. They could further activate caspases to break DNA of tumor cells and lead to tumor cell death (102–105). γδ T cells could kill the human squamous cell carcinoma through perforin and granzyme B (106). Perforin and granzyme B inhibitor significantly reduce the ability of Vγ9Vδ2 T cells to lyse breast cancer cells *in vitro* (107). Moreover, in patients with renal carcinoma, activated Vγ9Vδ2 T cells showed a strong cytotoxicity to autologous tumor cells through perforin and granzyme B (108).

Second, γδ T cells kill tumor cells through ADCC. The Fab and Fc segment of antibody could bind to the TAA and γδ T cells, respectively. Then γδ T cells are activated to kill the tumor cells. Upon interaction with tumor cells, the expression of CD16 (FcγRIIIA) could be up-regulated on γδ T cells to induce tumor death through ADCC (82, 109, 110). In chronic lymphocytic leukemia and breast cancer patients, the cytotoxicity of Vγ9Vδ2 T cells is significantly enhanced after treatment with monoclonal antibodies including rituximab, trastuzumab and alemtuzumab (111–113).

Third, γδ T cells kill tumors through the Fas/FasL pathway and TRAIL (106). FasL expressed on γδ T cells could bind to Fas, and formed Fas trimer, which lead to the binding of the death effector domain (DED) to Fas-associated death domain–containing protein (FADD), and then activate caspases to induced the apoptosis of target cells (114–116). Similar to Fas/FasL, TRAIL also activates caspases through FADD, and then leads to apoptosis of tumor cells (117–124). In addition, IFN-γ could enhance the cytotoxicity of γδ T cells by up-regulating the expression of Fas on osteosarcoma cells (125, 126).

Similar to Vγ9Vδ2 T cells, Vδ1 T cells could kill tumor cells through the perforin-granzyme B, Fas/FasL and TRAIL pathway (49, 50, 98, 101). For example, human skin Vδ1 T cells could secrete perforin to kill melanoma cells (127). Granzyme B^+^ Vδ1 T cells and TRAIL^+^ Vδ1 T cells showed strong cytotoxicity to lymphoma cells and chronic lymphocytic leukemia (128–130). Beyond that, *ex vivo* expanded Vδ1 T cells highly express FasL, and have strong cytotoxicity on colon cancer cells (131).

## γδ T Cells Enhance the Anti-Tumor Ability of Other Immune Cells

γδ T cells share similar functions as antigen presenting cells (APC), which could activate CD8^+^T cells (132, 133). When co-cultured with chronic myeloid leukemia (CML) cell lysates, the expression of co-stimulatory molecules (CD40, CD80 and CD86) and antigen-presenting molecule HLA-DR on Vγ9Vδ2 T cells could be strongly up-regulated. When these γδ T cells were co-cultured with CD8^+^ T cells, the proliferation rate of CD8^+^ T cells became 3 times faster than that of the control group (134, 135). Tumor cell fragments activate MAPK signaling pathways through Vγ9Vδ2TCR, up-regulate the expression of scavenger receptor CD36, enhance antigen uptake and processing of Vγ9Vδ2 T cells, and then induced tumor antigen-specific CD8^+^T cell response (136). Furthermore, γδ T cells toned to interact with cell surface-bound antibodies to acquire the ability of APC (137).

In addition, activated γδ T cells could secrete IFN-γ, which stimulates CSC to up-regulate the expression of MHC I molecules and CD54, and enhance the killing effect of CD8^+^T cells on tumor cells (138). Activated γδ T cells could also express CD137L to stimulate NK cells that upon proliferation exhibit strong anti-tumor activity through cell-to-cell contact (31).

**Figure 2:**
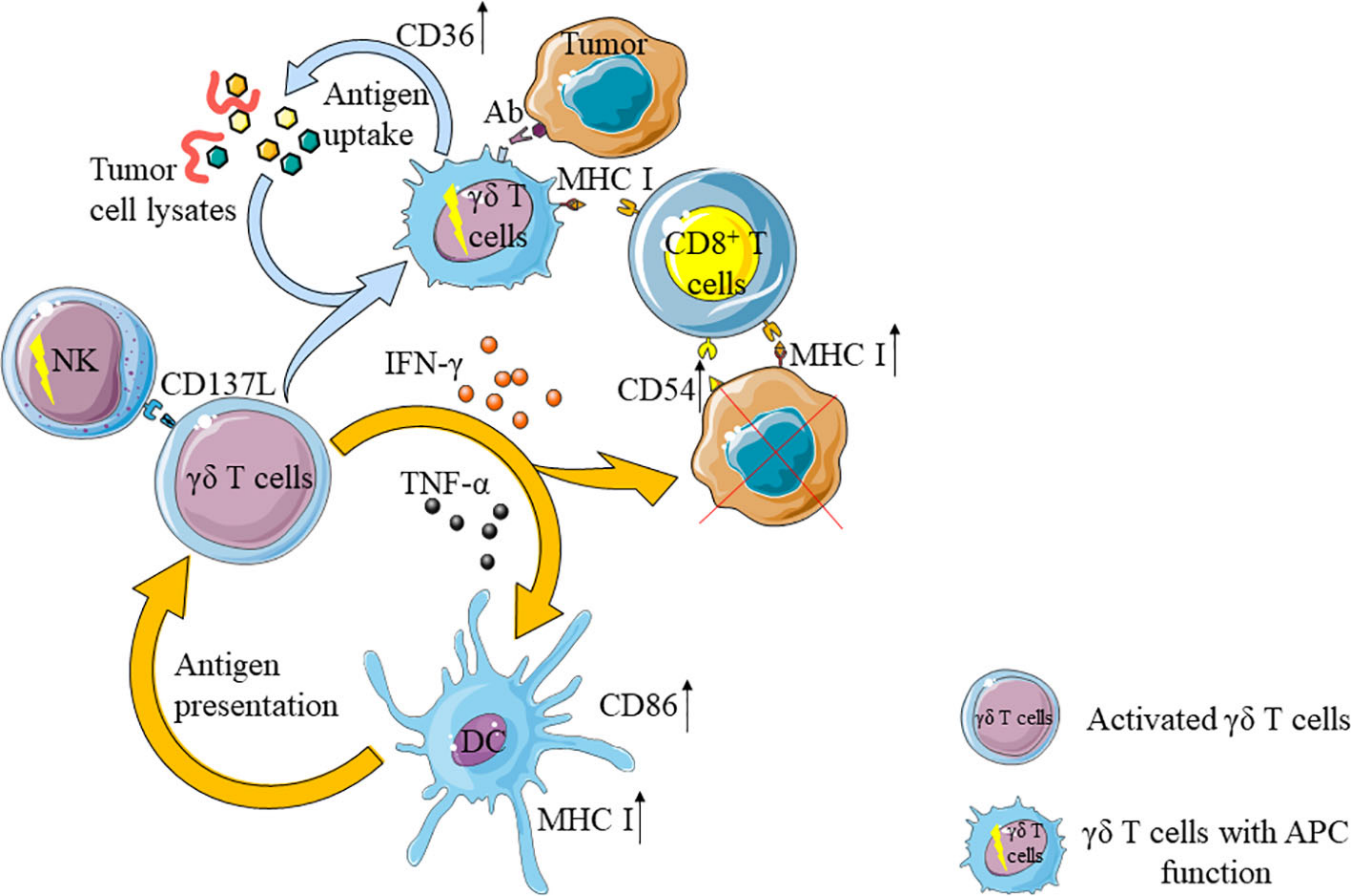
γδ T cells enhance the anti-tumor ability of other immune cells. On the one hand, activated γδ T cells can activate natural killer cells (NK) and dendritic cells (DC), activated DC can further activate γδ T cells. On the other hand, activated γδ T cells can up-regulate the expression of CD36 and enhance their antigen uptake ability after uptake of tumor cell lysates. At the same time, through contact with tumor cells with antibodies, the ability of antigen-presenting cells (APC) is obtained and CD8^+^T cells are activated. In addition, IFN- γ secreted by γδ T cells can up-regulate the expression of CD54 and MHC I molecules in tumor cells, and further enhance the anti-tumor effect of CD8^+^T cells.

The interaction between γδ T cells and dendritic cells (DC) is mutual. γδ T cells promote the maturation of DC, and mature DC induces the activation and proliferation of γδ T cells, which yield enhanced anti-tumor effect (139, 140). For example, activated Vγ9Vδ2 T cells could secrete IFN-γ and TNF-α to promote DC maturation and increase the expression of CD86 and MHC-I molecules on DC (141, 142). Mature DC could activate γδ T cells through presenting IPP, which synergizes with ATP-binding cassette transporter A1 (ABCA1), ApoA1 and BTN3A1 (143) (**Figure 2**).

## Tumor-Infiltrating γδ T Cells Promote Tumor Development by Secreting IL-17

Interestingly, patients with increased number of tumor-infiltrating γδ T cells have higher recurrence rates and likelihood of metastasis (144–146). Among the tumor-infiltrating γδ T cells, Vδ1 T cells are present as the main population and secrete IL-17 to promote tumor development. IL-17 can promote the proliferation of tumor cells by activating IL-6/STAT3 and NF-κB pathways. In addition, it can also stimulate tumor cells to secrete vascular endothelial growth factor (VEGF) and matrix* *metalloproteinases (MMP) to further help tumor metastasis. High levels of IL-17 have been found in patients with advanced tumor or metastasized tumors (64, 147, 148). For example, in patients with solid tumors, Vδ1 T cells account for a large proportion of tumor-infiltrating γδ T cells; unlike Vδ1 T cells in adjacent non-tumor tissues, tumor-infiltrating γδ T cells do not express granzyme B, perforin, IFN-γ, FasL, TRAIL and NKR, but secrete IL-17 (149–154). Majority of the tumor-infiltrating Vδ1 T cells were T_EM_ phenotype, while most of the Vδ1 T cells in healthy subjects were T_CM_ phenotype (64). Similarly and compared with healthy people, cancer patients have a larger proportion of Vδ1 T cells and higher IL-17 levels in their peripheral blood (155, 156).

## Tumor-Infiltrating γδ T Cells Inhibit the Anti-Tumor Function of Other Immune Cells

IL-17, secreted by tumor-infiltrating Vδ1 T cells not only acts on tumor cells directly, but can also recruit myeloid-derived suppressor cells (MDSC) to tumor (147, 148, 150). MDSC inhibits the activation of CD8^+^ T cells by expressing high levels of ARG1, which decomposes arginine in the tumor microenvironment (157–160).

In addition, tumor-infiltrating γδ T cells could significantly inhibit the maturation of CD4^+^ T cells (155). Studies in breast cancer settings showed that tumor-infiltrating γδ T cells could inhibit the maturation of CD4^+^ or CD8^+^ T cells and change their functions by arresting cell cycle in G0/G1 phase and increasing the expression of p53, P21, and P16. Through secreting IL-10 and TGF- β1, these suppressed T cells further amplified the inhibitory effect (161, 162). Beyond that, these cells express high levels of PD-L1 to promote the apoptosis of CD8^+^ T cells in cancer patients (41).

**Figure 3:**
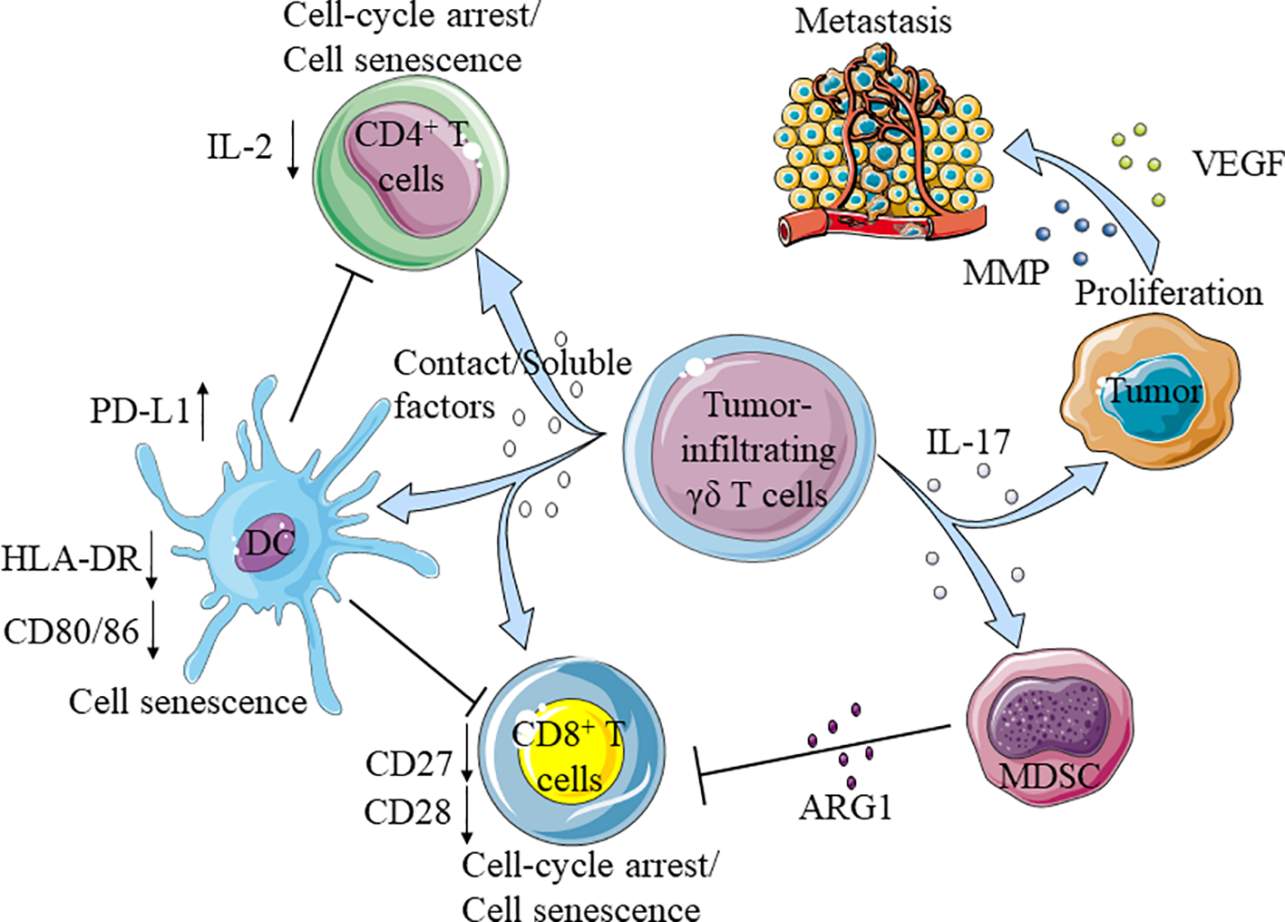
Tumor-infiltrating γδ T cells promote the development of tumor. Tumor-infiltrating γδ T cells secrete IL-17 promote the proliferation and metastasis of tumor cells, and recruit myeloid-derived suppressor cells (MDSC) to inhibit the function of CD8^+^T cells. Moreover, tumor-infiltrating γδ T cells directly impaired the function of CD4^+^/CD8^+^T cells and dendritic cells (DC), the aging DC further inhibited CD4^+^/CD8^+^T cells.

Vδ1 T cells could also inhibit the maturation of DC, reduce the expression of CD80/86 and HLA-DR on DC, attenuate the secretion of pro-inflammatory cytokines TNF- α, and up-regulate the expression of PD-L1 on the surface of DC (161, 163) (**Figure 3**).

## Anti-Tumor Immunotherapy With γδ T Cells

The unique antigen recognition mechanism of γδ T cells renders them the ability to kill various types of tumors. Therefore, γδ T cell-based therapies have been widely used in clinical as anti-tumor immunotherapies and achieved good results (**Table 2**). At present, the most routine method in these therapies is to activate the anti-tumor activity of natural γδ T cells and the Vγ9Vδ2 T cells, which as the most abundant subtype in peripheral blood are often selected and utilized through transferring back to cancer patient after stimulation *in vitro* or direct activation *in vivo*.

**Table 2:** Clinical trials of γδ T cell-based immunotherapy.

Cell types	Cell source	Stimulation method	Disease	Number of patients	Phase	Ref.
Vγ9Vδ2 T	Peripheral blood of healthy people	ZOL+ a variety of interleukins (Patent pending)	Cholangio-carcinoma	1	IV	(52)
Vγ9Vδ2 T	Peripheral blood	ZM3B1PP+IL-2	Advanced renal cell carcinoma	7	Pilot study	(164)
Vγ9Vδ2 T	Peripheral blood	2M3B1PP+ZOL+IL-2	Advanced renal cell carcinoma	11	I/II	(165)
Vγ9Vδ2 T	Peripheral blood	ZOL+IL-2	Several solid tumors	18	I	(166)
Vγ9Vδ2 T	Peripheral blood	ZOL+IL-2	Multiple myeloma	6	I	(167)
Vγ9Vδ2 T	Peripheral blood	IPH1101+IL-2	Metastatic renal cell carcinoma	10	I	(168)
Vγ9Vδ2 T	Peripheral blood	ZOL+IL-2	Recurrent non-small-cell lung cancer	10	I	(169)
Vγ9Vδ2 T	Peripheral blood	ZOL+IL-2	Advanced non-small lung cancer	15	I	(170)
Vγ9Vδ2 T	Peripheral blood	ZOL+IL-2	Malignant ascites (gastic cancer)	7	Pilot study	(171)
Vγ9Vδ2 T	Peripheral blood	ZOL+IL-2	Refractory renal cell carcinoma	12	Pilot study	(172)
		Injection ZOL+IL-2	Neuroblastoma	4	I	(173)
		Injection ZOL+IL-2	Several advanced tumors	21	I/II	(174)
		Injection ZOL+IL-2	Lymphoid malignacies	19	Pilot study	(175)
		Injection IPH 1101+IL-2	Several solid tumors	28	I	(176)

The most widely used stimulants for expanding Vγ9Vδ2 T cells *in vitro* are zoledronic acid (ZOL) and IL-2. As a kind of bisphosphate, ZOL could specifically inhibit farnesyl pyrophosphate synthase (FPPS) in isoprene biosynthesis pathway, thus causing the accumulation of endogenous PAg in cells and promoting the activation of Vγ9Vδ2 T cells (65). This method could effectively expand and activate Vγ9Vδ2 T cells from patients or healthy people *in vitro* (52). In addition, another kind of PAg, 2-methyl-3-butenyl-1-pyrophosphate(2M3B1-PP) could also effectively stimulate and expand Vγ9Vδ2 T cells (164, 165). The activated immune cells are transferred back into the patients to produce anti-tumor effects. In order to track the activated Vγ9Vδ2 T cells, they are typically labeled with indium^111^. Studies have confirmed that these cells mainly metastasize to the lung, liver and spleen, as well as to the tumor sites (166). In the treatment of patients with multiple myeloma, the stimulated Vγ9Vδ2 T cells expressed high levels of NKG2D and IFN-γ, but not IL-17. After treatment, the number of Vγ9Vδ2 T cells in the tumor microenvironment increased, lasting as long as 4 weeks (167). In patients with renal cell carcinoma and non-small cell lung cancer, repeated injections of IL-2 has demonstrated good safety (168), enhanced the cytotoxicity of Vγ9Vδ2 T cells. As results, the deterioration of tumor was alleviated with patients’ condition stabilized, and the survival time was pro-longed (164, 165, 169, 170). In the clinical study of malignant ascites, after transferring back the activated Vγ9Vδ2 T cells, the number of tumor cells in ascites decreased significantly and the level of IFN-γ in ascites increased. During the course of treatment, there were no significant adverse effects and the amounts of ascites decreased significantly (171). In addition to the direct anti-tumor effect of Vγ9Vδ2 T cells, the numbers of CD4^+^ T and CD8^+^ T cells could also get increased after the allogeneic Vγ9Vδ2 T cells were transferred into the patients, as shown in another study of cholangio-carcinoma (52).

Vγ9Vδ2 T cells could also be activated *in vivo*. Injection of ZOL and IL-2 could directly activate these cells in cancer patients. Serval clinical trials have demonstrated that, upon injection and activation of Vγ9Vδ2 T cells *in vivo*, IFN-γ was strongly induced and the deterioration of the tumor was controlled (53, 172–175). Besides ZOL, Vγ9Vδ2 T cells could also be activated by synthetic PAg or bromohydrin pyrophosphate (BrHPP, IPH1101) with good safety tolerability in patients (176).

## Optimization of γδ T Cell-Based Immunotherapy

In clinic, repeated use of ZOL and IL-2 carry the liability in inhibiting the proliferation of Vγ9Vδ2 T cells (172), which could be alleviated by vitamin C (VC) and L-ascorbic acid 2-phosphate (pVC). VC has the ability to reduce the apoptosis of γδ T cells during stimulation, and pVC may enhance the expansion of γδ T cells. Therefore, VC and pVC have been utilized to improve the efficacy of the γδ T cells in anti-tumor immunotherapy (177). In addition, cytotoxicity of Vγ9Vδ2 T cells could be improved by adding IL-21, IL-15, or IL-33 *in vitro* (55, 178–182). Anti-cancer drug Gemcitabine or anti-epileptic drug Valproic acid (VPA) in combination with ZOL could also enhance the anti-tumor ability of γδ T cells (183, 184).

In recent years, chitosan nanoparticles (CSNPs) and antibodies have been developed as potential anti-tumor therapies. CSNPs have been shown to regulate γδ T cells by up-regulating the expression of NKG2D, CD56 and FasL, and enhancing their anti-tumor functions (185). TIM-3 could also inhibit the killing effect of Vγ9Vδ2 T cells on tumor by reducing the expression of perforin and granzyme B. PD-1 and TIM-3 antibodies could protect anti-tumor activity of Vγ9Vδ2 T cells (186–188). Beyond these, the application of bispecific antibodies can also promote γδ T cells to inhibit tumor development. For example, in the study of hepatoblastoma and pediatric hepatocellular carcinoma, the application of EpCAM/CD3-bispecific BiTE antibody (MT110) enhanced the anti-tumor ability of γδ T cells; similarly, in epithelial ovarian cancer and pancreatic ductal adenocarcinoma, bispecific antibody [HER2xCD3] and [(HER2)2xVγ9] (tribody format) could also effectively enhance the cytotoxicity of γδ T cells (189–193).

Finally, chimeric antigen receptor engineered γδ T (CAR-γδ T) technology is another new direction in immunotherapy. CAR- γδ T cells could target GD2, a TAA on the surface of neuroblastoma cells, and effectively kill tumors. This kind of CAR-γδ T cells need to recognize GD2 to become activated. Such mechanism ensures the specificity of these cells in killing tumor cells and offering lower toxicities and side effects (194, 195). On the hand, Vγ9Vδ2TCR could also be transduced into αβ T cells. These CAR-T cells are called TEGs, which carry not only the extensive recognition ability of γδ T cells but also and the memory ability of αβ T cells (196–199).

## Summary

Taken together, we described in this review that Vδ1 T cells and Vγ9Vδ2 T cells are the two most important subgroups of human γδ T cells. Peripheral Vδ1 T cells and Vγ9Vδ2 T cells could recognize tumor cells through TCRγδ and NKR, and kill them through perforin-granzyme B, Fas/FasL and TRAIL. Activated Vγ9Vδ2 T cells could perform the function of APC, and furthermore, they could activate NK cells and DC directly. On the contrary, tumor-infiltrating Vδ1 T cells promoted tumor development by secreting IL-17 and inhibiting the maturation of CD4^+^/CD8^+^ T cells and DC. In immunotherapy, ZOL, 2M3B1-PP or IPH1101 has been commonly used to activate Vγ9Vδ2 T cells to achieve anti-tumor effect. The failure caused by repeated application of this method can be solved by adding VC or replacing cytokines. In addition, new classes of drugs such as CSNPs, were also applied to γδ T cell-based anti-tumor immunotherapy.

It is noteworthy to mention that although Vδ1 T cells account for the majority of tumor-infiltrating γδ T cells, the definition of γδ T cell subsets still rely on their profile in cytokine production (32, 64, 149). Secondly, the interaction mechanism between γδ T cells and the environment or other immune cells remains to be further elucidated. For example, Vγ9Vδ2 T cells could ingest LDL-cholesterol upon activation and lead to reduced function, suggesting that obesity may inhibit the anti-tumor activity of γδ T cells (200). Another myth exists in how exactly soluble molecules mediate the inhibition of γδ T cells in tumor microenvironment (161, 163). In addition, γδ T cell-based immunotherapy needs to be further optimized, with emphasis on how to carry out personalized therapy according to the actual situation of individual patient.

In summary, a more comprehensive understanding of the biological characteristics of γδ T cells, an important group of lymphocytes, will guide the improvement of their clinical application methods and provide new strategies to fight against human cancers.

## Author Contributions

JZ completed the writing of the classification of γδ T cells. XC and XW completed the writing of introduction and summary. YL and GL completed the writing of the rest of this review. All authors contributed to the article and approved the submitted version.

## Funding

Funding support from China Postdoctoral Science Foundation (2018M641666).

## Conflict of Interest

The authors declare that the research was conducted in the absence of any commercial or financial relationships that could be construed as a potential conflict of interest.
